# iNKT Cells in Secondary Progressive Multiple Sclerosis Patients Display Pro-inflammatory Profiles

**DOI:** 10.3389/fimmu.2016.00555

**Published:** 2016-11-30

**Authors:** Sara De Biasi, Anna Maria Simone, Milena Nasi, Elena Bianchini, Diana Ferraro, Francesca Vitetta, Lara Gibellini, Marcello Pinti, Cinzia Del Giovane, Patrizia Sola, Andrea Cossarizza

**Affiliations:** ^1^Department of Surgery, Medicine, Dentistry and Morphological Sciences, University of Modena and Reggio Emilia, Modena, Italy; ^2^Neurology Unit, Department of Biomedical, Metabolic and Neurosciences, Nuovo Ospedale Civile Sant’Agostino Estense, University of Modena and Reggio Emilia, Modena, Italy; ^3^Department of Life Sciences, University of Modena and Reggio Emilia, Modena, Italy; ^4^Department of Diagnostic and Clinical Medicine and Public Health, University of Modena and Reggio Emilia, Modena, Italy; ^5^Department of Medical and Surgical Sciences of Children and Adults, University of Modena and Reggio Emilia, Modena, Italy

**Keywords:** multiple sclerosis, iNKT cells, cytokines, flow cytometry, immunomodulatory drugs

## Abstract

**Background:**

Multiple sclerosis (MS), an autoimmune disease with neurodegeneration and inflammation is characterized by several alterations of different T cell subsets. However, few data exist on the role of iNKT lymphocytes.

**Objective:**

To identify possible changes in the phenotype of iNKT cells in patients with different clinical forms of MS and find alterations in their polyfunctionality [i.e., ability to produce simultaneously up to four cytokines such as IL-17, tumor necrosis factor (TNF)-α, interferon (IFN)-γ, and IL-4].

**Methods:**

We studied a total of 165 patients, 91 with a relapsing–remitting form [RR; 31 were treated with interferon (IFN)1a-β, 25 with natalizumab (NAT), 29 with glatiramer acetate; 17 were newly diagnosed RR without treatment, 19 not-active RR without treatment]. Forty-four patients had a progressive MS: 20 primary progressive (PP) and 24 secondary progressive (SP). A total of 55 age- and sex-matched subjects represented healthy controls (CTR). Among fresh peripheral blood mononuclear cells, iNKT cells were identified by flow cytometry. Moreover, the capability of iNKT cells to produce different cytokines (IL-17, TNF-α, IFN-γ, and IL-4) after *in vitro* stimulation were evaluated in 18 RR (11 treated with NAT and 7 with IFN), 4 PP, 6 SP, and 16 CTR.

**Results:**

No main differences were found in iNKT cell phenotype among MS patients with different MS forms or during different treatments. However, the polyfunctional response of iNKT cells showed Th1 and Th17 profiles. This was well evident in patients with SP form, who are characterized by high levels of inflammation and neurodegeneration, and exhibited a sustained increase in the production of Th17 cytokines. Patients treated with NAT displayed lower levels of iNKT cells producing IL-17, TNF-α, and IFN-γ.

**Conclusion:**

Our data suggest that the progressive phase of the disease is characterized by permanent iNKT activation and a skewing towards an inflammatory phenotype. Compared to other treatments, NAT was able to modulate iNKT cell function.

## Introduction

Multiple sclerosis (MS) is a chronic inflammatory and neurodegenerative disease of the central nervous system (CNS). The pathogenesis of MS is still unknown, but it is generally accepted that the alteration of autoimmune homeostasis plays a major role. The relationship between inflammation and neurodegeneration and their contribution to the different phases of the disease remains ambiguous (1). It is also unclear how these two processes are generated and controlled by different components of the immune system. Although adaptive immunity is clearly involved in the pathogenesis of MS, there is increasing evidence on the role of cells belonging to innate immunity (2).

iNKT cells belong to the family of innate-like lymphocytes; they are T lymphocytes characterized by the expression of NK cell markers and an invariant alpha chain and represent less than 1% of circulating T lymphocytes (3). iNKT cells are a specialized lymphocyte subset, which expresses an invariant Vα24Jα18 T-cell receptor and recognizes as cognate antigens self and foreign lipids presented by CD1d (4, 5). On the basis of CD4 and CD8 expression, mature iNKT cells can be divided into functionally distinct subsets, i.e., CD4+CD8−, CD4−CD8−, and CD4−CD8+ (3). iNKT cells mediate both protective and regulatory immune functions through a rapid production of cytokines (6). Indeed, activated iNKT cells can rapidly and simultaneously release large amounts of Th1 [interferon (IFN)-γ, tumor necrosis factor (TNF)-α] and Th2 cytokines [interleukin (IL)-4, IL-12, and IL-13] (7), and can also produce, under certain conditions, IL-10 (8) and Th17 cytokines such as IL-17 and IL-22 (9).

The aforementioned different subsets of iNKT cells have different immunological properties: CD4+ iNKT cells release Th1 and Th2 cytokines, while CD8+ and CD4−, CD8− cells exhibit Th1 phenotypes and cytotoxic activity (10). Moreover, a regulatory phenotype of iNKT cells has also been described, based upon the production of IL-10 and transforming growth factor-beta, and on the expression of classical markers of regulatory T cells such as CD4, high expression of CD25, FoxP3, and the lack of CD127 (11). Of note is also that iNKT cells are able to produce granulocyte-macrophage colony-stimulating factor (12), which induces migration of human monocytes across the blood–brain barrier (13).

It has been suggested that iNKT cell defects can cause failure of immune regulation that predisposes an individual to autoimmune diseases (14–16). For example, the decreased frequency of iNKT cells in non-obese diabetic mice initially suggested the regulatory role of this cell subset (17). Similarly, in patients with systemic lupus erythematosus (SLE), the percentage of iNKT cells was lower than that of healthy subjects (18). In addition, many studies confirmed that NKT cells are characterized by reduced function and proliferative potential in rheumatoid arthritis (RA) and that cytokine production by NKT cells could depend from different backgrounds and at different stages of RA (19). Previous studies on iNKT cells in patients affected by MS showed the presence of different defects, but yielded contradictory results, mainly because of non-stringent methods used for the identification of iNKT cells that are quite rare in blood, and of the limited analysis of the distribution and/or function of their subsets (3, 20–22).

There is a general agreement that complex defects of innate cells exist in this pathology (3, 23), but the role of iNKT cell defects in MS has not been fully elucidated, and a detailed characterization of the phenotype and functionality of iNKT cells in the different forms of MS has never been reported. For these reasons, using the most advanced techniques based upon high-speed polychromatic acoustic flow cytometry, we evaluated the amount of these cells in patients with different forms of MS and during treatments with different immunomodulatory drugs. Moreover, we characterized iNKT phenotype and functional activities, i.e., the ability of different iNKT cell subsets of producing cytokines, and we correlated immunological data with clinical parameters.

## Materials and Methods

### Patient Selection

Patients were selected at the “Center for Demyelinating Diseases” of the Neurology Unit of Modena (Nuovo Ospedale Civile S. Agostino Estense, Modena, Italy). Evaluation of entry criteria and subsequent enrollment in the study occurred during patients’ planned routine visits. Inclusion criteria were: age between 18 and 65 years and diagnosis of MS according to the 2010 revised McDonald Criteria (24). Patients with concomitant infections (viral or bacterial), with a relapse treated with steroids in the preceding 30 days, and patients with non-steroidal anti-inflammatory drug intake within the preceding 48 h, were excluded.

A total of 165 MS patients were selected for the study:

– 85 RR patients treated with disease-modifying drugs for at least 6 months: 31 with β-interferon 1a (IFN1a-β) 29 with glatiramer acetate (GA) and 25 with natalizumab (NAT);
– 44 untreated patients with a progressive form of the disease: 20 with PP, and 24 with secondary progressive (SP) form, without superimposed relapses in the preceding 2 years;
– 17 newly diagnosed, untreated RR patients. This group was classified as “active” RR (ARR), following the 2013 revisions (25) of the previous MS phenotype descriptions. In particular, activity was determined by clinical relapses and/or MRI activity (contrast-enhancing lesions; new/enlarging lesions on T2-weighted images) in the preceding year;
– 19 RR patients, untreated for at least 6 months prior to enrollment, and without clinical or MRI activity in the preceding 2 years. This group was classified as “not-active” RR (NARR) (25).

The functional analysis was carried out on 57 patients belonging to the abovementioned groups, in particular: 13 RR patients treated with NAT, 11 with GA, 12 with IFN; 5 patients with not-active RR, 4 with PP, 12 with SP, and 26 controls (CTR).

For each patient, the following parameters were recorded: demographic (sex, age), clinical [age at onset, disease duration, disability measured by Kurztke’s Expanded Disability Severity Score – EDSS (26), delta-EDSS in the preceding year, number of relapses in the preceding year, MS Severity Score – MSS (27), and MRI data (date of previous MRI assessment, number of gadolinium-enhancing lesions, and/or new/enlarging T2 lesions)].

Fifty-five sex- and age-matched healthy subjects, without a history of autoimmune diseases or immunosuppressant/corticosteroid therapy, were chosen as controls. In order to avoid any potential effect of aging on iNKT cells, we divided controls in two groups: 39 younger controls (YCTR, mean age ± SE 34.7 ± 1.1 years) and 16 older controls (OCTR, 53.8 ± 1.5 years): YCTR were compared to ARR patients, while OCTR were compared to NARR and progressive patients who are older than RR patients.

The study obtained the Ethical Committee’s approval (no. 246/12) of the province of Modena (Italy), and, at the time of enrollment, all individual participants included in the study signed the written informed consent in accordance with the Declaration of Helsinki. Table 1 summarizes MS patients’ characteristics.

**Table 1:** **Clinical characteristics of multiple sclerosis patients**.

	Total	Newly diagnosed RR (ARR)	Not-active RR (NARR)	RR treated with IFN	RR treated with GA	RR treated with NAT	SP	PP
No. of patients	165	17	19	31	29	25	24	20
Males, *n* (%)	43 (26)	6 (35)	4 (21)	8 (25)	6 (21)	4 (16)	6 (25)	9 (45)
Females, *n* (%)	122 (74)	11 (65)	15 (79)	23 (75)	23 (79)	21 (84)	18 (75)	11 (55)
Age, years^a^ range (min; max)	45.1 ± 11.6 (19; 66)	35.5 ± 8.7 (21; 52)	51.1 ± 7.7 (40; 64)	42.9 ± 8.4 (24; 58)	40.7 ± 8.2 (29; 56)	35.5 ± 9.7 (19; 51)	55.7 ± 7.9 (38; 66)	57.2 ± 7.4 (47; 65)
Age at onset, years^a^	33.8 ± 10.1	33.7 ± 9	31.4 ± 9.5	34 ± 8.4	34 ± 9.3	27.9 ± 7.9	34 ± 10.2	43.1 ± 11.7
Disease duration (months)^b^	135.7 ± 107.7	24.5 ± 23.5	237.4 ± 98.5	105.8 ± 78.1	79.4 ± 58.2	90.3 ± 52.7	259.1 ± 104.4	166.7 ± 94.1
Number relapses preceding year^a^	0.2 ± 0.6	0.9 ± 0.6	0.1 ± 0.2	0.1 ± 0.3	0.1 ± 0.4	0.6 ± 1.2	0	0
Severity score^a^	3.2 ± 2.9	2.8 ± 2.7	0.6 ± 0.5	1.6 ± 1.6	2 ± 2.1	3.4 ± 2.5	6.4 ± 2.2	6.2 ± 2.6
EDSS^a^	2.6 ± 2.4	1.3 ± 1.1	1.1 ± 0.7	1.3 ± 1.4	1.1 ± 0.9	2.1 ± 1.3	6.4 ± 1.2	5.2 ± 2
Delta – EDSS (preceding 12 months)^a^	0.1 ± 0.4	2.1 ± 1.3	0 ± 0.3	0 ± 0.4	0 ± 0.1	0 ± 0.4	0.2 ± 0.2	0.2 ± 0.4

*^a^Values expressed as mean ± SD*.

*^b^Values expressed as median ± SD*.

### Isolation of Peripheral Blood Mononuclear Cells and Polychromatic Flow Cytometry

Thirty milliliters of venous blood were collected from each subject into ethylenediaminetetraacetic acid (EDTA) tubes. First, we performed the identification and enumeration of CD3+ T lymphocytes in whole blood samples using the “CD3 easy count kit,” a kit with no lyse–no wash reagents for the volumetric absolute counting of CD3+ T cells, and the CyFlow Counter (Partec GmbH, Münster, Germany). Then, peripheral blood mononuclear cells (PBMCs) were isolated by Ficoll–Hypaque density gradient according to standard procedures and immediately processed for immunophenotypic analysis.

Freshly isolated PBMCs were stained Aqua Live Dead Probe (Thermofisher) and with mAbs conjugated with the appropriate fluorochrome at previously defined optimal concentrations. The following mAbs were used: anti-Vα24JαQ TCR chain PE (clone 6B11 was used because it reacts with a unique determinant in the CDR3 region of the invariant Vα24-JαQ TCR chain), anti-CD4 APC-H7 (clone RPA-T4), anti-CD8 APC (clone RPA-T8), anti-CD161 FITC (clone DX12), anti-CD3 PE-Cy7 (clone UCHT1) [all from Becton Dickinson Biosciences (BD), San José, CA, USA]; anti-CD19 ECD (clone J3-119) and anti-CD14 ECD (clone RMO52) (from Beckman Coulter Inc., Hialeah, FL, USA). PBMCs were incubated for 20 min at room temperature, washed with phosphate buffered saline, and immediately analyzed. A minimum of 10 million cells per sample were acquired and analyzed as described below.

### *In Vitro* Cytokine Production by iNKT Cells

For functional assays, freshly isolated PBMCs were stimulated for 4 h at 37°C in a 5% CO_2_ atmosphere with phorbol 12-myristate 13-acetate (PMA) (200 ng/mL, Sigma Aldrich, Saint Louis, MO, USA) plus ionomycin (1 mg/mL, Sigma-Aldrich), in complete culture medium (RPMI 1640 supplemented with 10% fetal bovine serum and 1% each of l-glutamine, sodium pyruvate, non-essential amino acids, antibiotics, 0.1 M HEPES, 55 μM β-mercaptoethanol). For each sample, at least 5 million cells were left unstimulated as negative control and at least 15 million cells were stimulated. All samples were incubated with a protein transport inhibitor containing brefeldin A (Golgi Plug, BD). After 4 h of stimulation, cells were stained with live–dead AQUA (Thermo Fisher Scientific) and surface mAbs recognizing Vα24JQ TCR chain PE, CD3 PE-Cy5, CD4 AF700, and CD8 APC-Cy7 (Biolegend, San Diego, CA, USA). Cells were washed with stain buffer (BD) and fixed and permeabilized with the cytofix/cytoperm buffer set (BD) for cytokine detection. Then, cells were stained with mAbs recognizing IL-17 BV421 (clone BL168), TNF-α BV603(clone MAb11), IFN-γ FITC (clone B27), or IL-4 APC [clone 8D4-8 (all mAbs from Biolegend)], using previously described strategies for intracellular cytokine detection (28).

Up to 20 million cells per sample were acquired by an acoustic focusing flow cytometer (Attune NxT, Thermo Fisher Scientific, Eugene, OR, USA), equipped with four lasers for excitation at 405, 488, 561, and 638 nm. We used the recently described the strategy required for the cytofluorimetric identification of rare events (24). Data were acquired in list mode using Attune NxT 2.1 software, and analyzed by FlowJo 9.8.5 (Tree Star Inc., Ashland, OR, USA) under Mac OSX. Samples were compensated by software, after acquisition. Single staining and fluorescence minus one (FMO) controls were performed for all mAbs of the panel to set proper compensation and define positive signals.

### Statistical Analysis

Quantitative variables were compared with non-parametric Mann–Whitney *U*-test. The correlations between clinical parameters and molecular data were performed by Spearman Correlation test and linear regression analysis. A *p* Value <0.05 was considered significant, except in case of multiple analyses, when the Bonferroni correction was applied. Data are represented as the mean ± SEM. Statistical analyses were performed using Prism 6.0 (Graphpad Software Inc., La Jolla, San Diego, CA, USA).

Simplified Presentation of Incredibly Complex Evaluation (SPICE) software (version 5.3, kindly provided by Dr. Mario Roederer, Vaccine Research Center, NIAID, NIH, Bethesda, MD, USA) was used to analyze polychromatic flow cytometry data, as described (29, 30). For the analysis of iNKT cell functionality, a threshold of 0.05% was set on the basis of the distribution of negative values (31, 32).

## Results

### Identification of iNKT Cells as Rare Events

**Figure 1:**
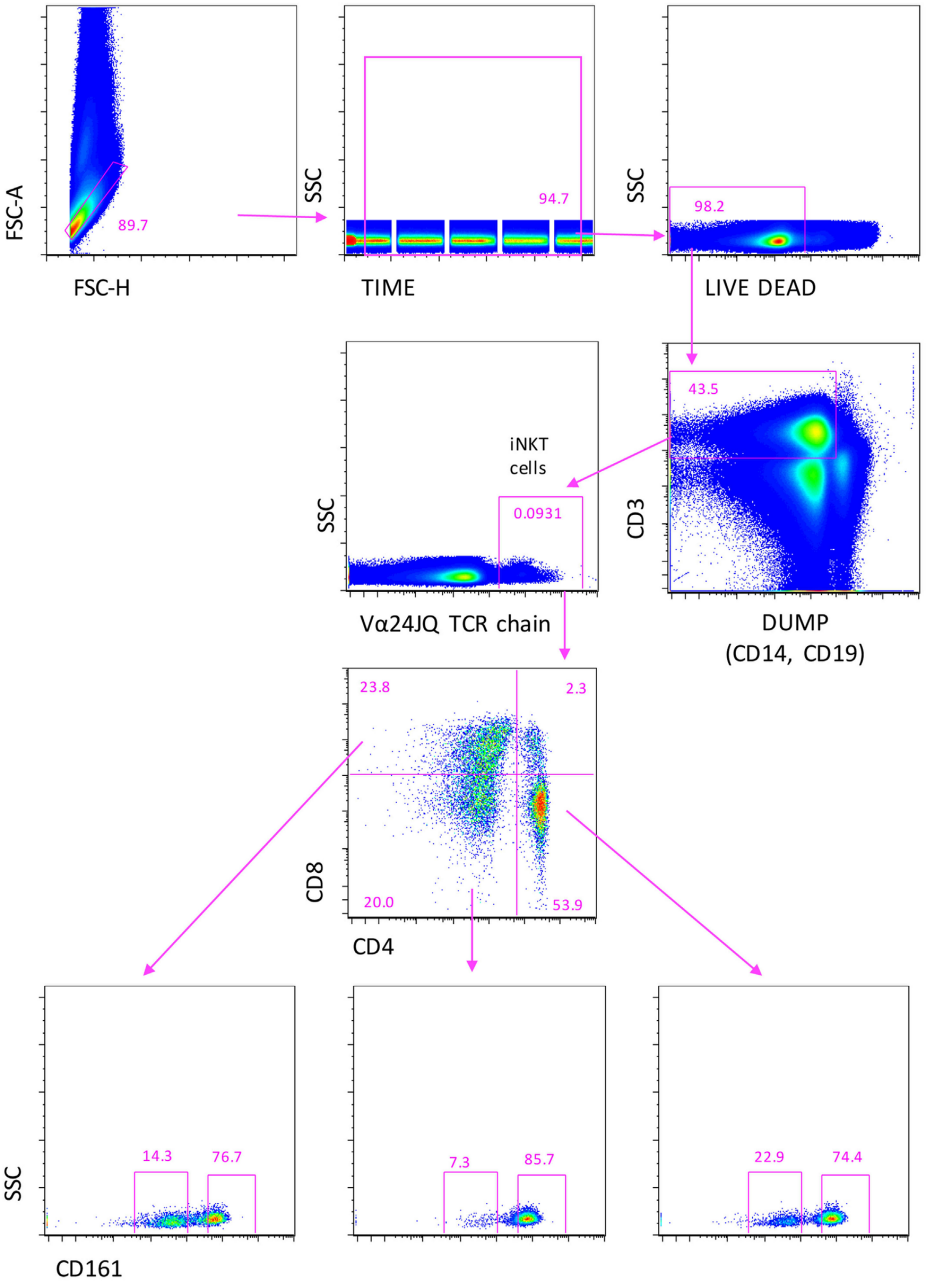
**Gating strategy to identify iNKT cells expressing CD4, or CD8, or double negative (DN) cells**. A first gate was set on FCS-height vs. FCS-area to eliminate doublets and aggregates, the on “time of acquisition,” then, on living cell, according to negativity to live–dead staining. CD3+ T lymphocytes were identified after the removal of monocytes (CD14+) and B cells (CD19+), recognized in the DUMP channel. Then, iNKT cells were recognized according the positivity to Vα24-JαQ TCR chain. Finally, CD4+, CD8+, or CD4−, CD8− double negative (DN) iNKT were identified. In all these subsets, the expression of CD161 was evaluated. Seven color flow cytometry was used, and a minimum of 10 million events per sample were acquired using Attune NxT flow cytometer.

In order to identify circulating iNKT whose percentage in peripheral blood is typically much less than 1%, it is mandatory to acquire a huge number of cells (28, 33). Thus, for the phenotypic and functional analysis, we made use of a novel acoustic flow cytometer able to align cells in the flow chamber by using ultrasounds and to acquire up to 35,000 events/second. This was crucial to obtain the number of cells, which is required for a correct statistical analysis, which was always >5 millions. The gating strategy used to identify iNKT cells with their subsets is shown in Figure 1, related to 22 million cells. Singlets were selected according to FSC-A and FSC-H, and the initial part of the acquisition was removed to avoid any possible instability of the flux. A gate was set to identify living cells, i.e., those negative to the staining with the live–dead probe. Next, T cells were identified by positivity for CD3 expression and negativity for CD14 and CD19, markers included into the DUMP channel. iNKT cells were selected according to the presence of a Vα24-JαQ TCR chain. On the basis of the expression of CD4 and CD8, different subsets of iNKT cells were identified: CD4+, CD8−, or CD4−, CD8+, or CD4−, CD8−. In all these subpopulations, the expression of CD161 was evaluated.

### Phenotype of iNKT Changes in Different Forms and during Different Treatments of Multiple Sclerosis

**Figure 2:**
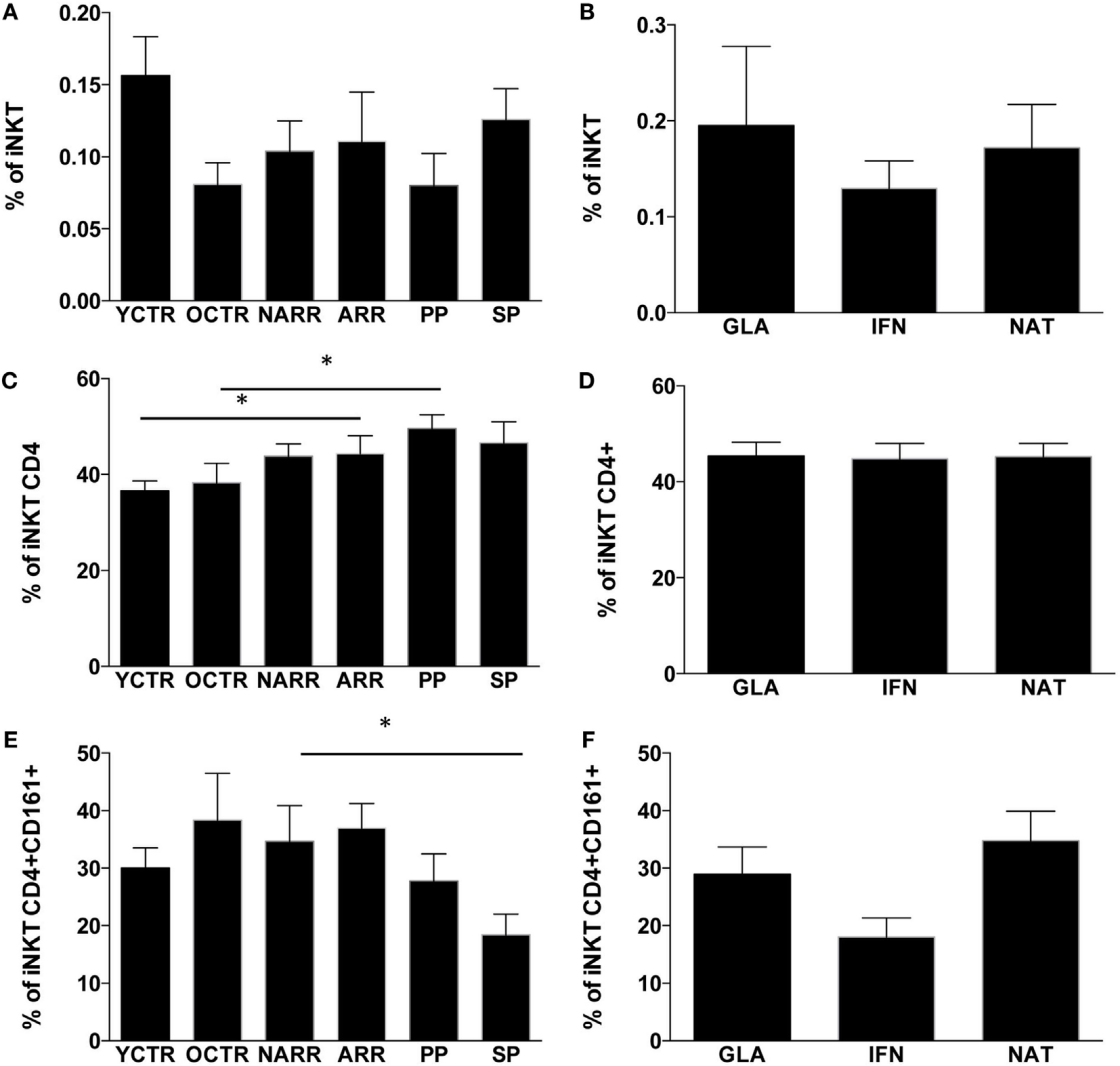
**Percentages of iNKT cells and their subpopulations among the different groups of MS patients and controls**. **(A)** Percentage of iNKT cells among patients with different multiple sclerosis form; **(B)** percentage of iNKT cells among RR patients treated with different immunomodulatory drugs; **(C)** percentage of CD4+ iNKT cells among patients with different multiple sclerosis forms; **(D)** percentage of CD4+ iNKT cells among RR patients treated with different immunomodulatory drugs; **(E)** percentage of CD4+ iNKT cells expressing CD161+ among patients with different multiple sclerosis forms; **(F)** percentage of CD4+ iNKT expressing CD161 cells among RR patients treated with different immunomodulatory drugs. ARR, active relapsing–remitting; NARR, untreated patients with not-active RR; PP, untreated primary progressive; SP, secondary progressive; GLA: RR patients who had been treated for at least 6 months with either glatiramer acetate (GLA), or with beta-interferon-1a (IFN) or with natalizumab (NAT). YCTR: donors whose age was <46 years; OCRT: controls >46 years. Quantitative variables were compared with non-parametric Mann–Whitney *U*-test. *p Values <0.05 were considered statistically significant. Data are represented as the mean ± SEM.

The percentage of T lymphocytes and their absolute number were similar among all groups of patients, but those treated with NAT showed a higher number of T lymphocytes if compared to other treated patients (data not shown). Similarly, the percentage and the absolute number of total iNKT cells were similar among different forms of MS (Figure 2A) or among RR patients treated with different immunomodulating therapies (Figure 2B).

As far as different subpopulations of iNKT cells are concerned, ARR patients and PP patients displayed higher levels of CD4+ iNKT cells if compared to their CTR group (Figure 2C). No differences were found among RR patients taking different treatments. SP patients displayed lower levels of CD4+ iNKT cells expressing CD161 if compared to not-active RR patients (Figure 2D), and higher levels of the same cell populations were found in RR patients treated with NAT (Figure 2E). Patients treated with IFN showed a reduction of CD4+ iNKT cells expressing CD161 (Figure 2F), even if the difference did not reach statistical significance.

### Cytokine Production by Different Subsets of iNKT Cells

In order to study the polyfunctionality of iNKT cell subpopulations, in a large set of experiments, we stimulated cells *in vitro* with a massive stimulus, represented by PMA plus ionomycin. However, before starting these experiments, we had analyzed polyfunctionality in iNKT cell lines, which were generated either from healthy donors or MS patients. We found that after a few weeks of culture in the presence of required stimuli such as high amounts of IL-2, autologous feeder layers and α-galactosylceramide (a synthetic glycolipid derived from a marine sponge that is the most efficient compound for activating the majority of NKT cells), all cells, both from patients and controls, presented the same phenotype, i.e., >50% DN, about 33% CD8+, and few CD4+; almost all cells were CD161++. For this reason, we chose to analyze phenotypic and functional changes after a short period of incubation, i.e., 4 h of stimulation being well aware that PMA plus ionomycin was a massive stimulus, also able to induce cell death (in all samples, we used a viability marker to eliminate dead cells from the analysis).

**Figure 3:**
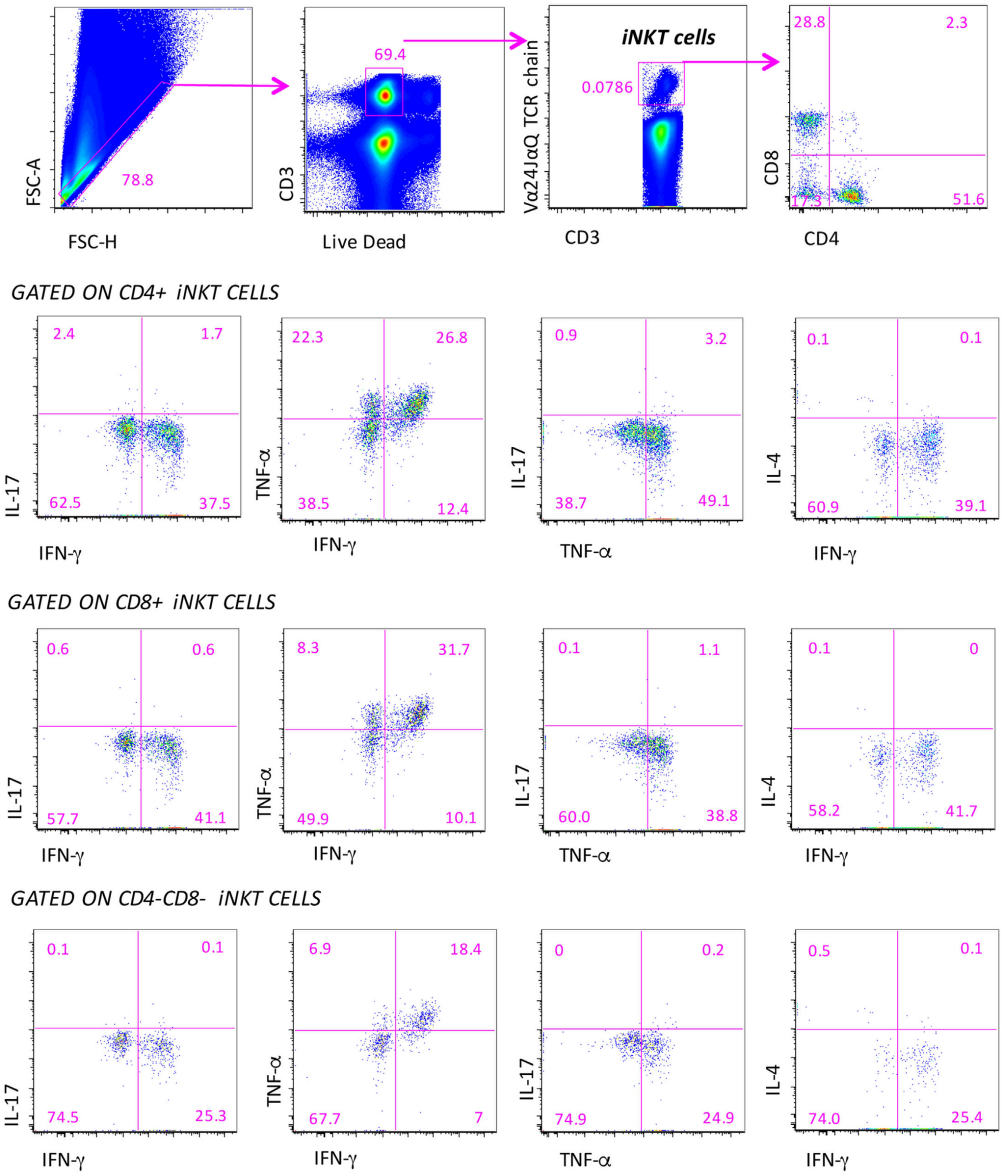
**Gating strategy for the identification of the polyfunctionality of different subpopulations of iNKT cells**. Several electronic gates were set, first to exclude doublets on the basis of physical parameters (forward scatter area and height), then to exclude dead cells and to identify CD3+ T lymphocytes, and finally to recognize iNKT cells and their subpopulations, on the basis of CD4 and CD8 expression. Among these subpopulations, the production of IL-4, IL-17, TNF-α, and IFN-γ is quantified. Nine-color flow cytometry was used, acquiring a minimum of 10 million events per sample.

In a representative group of MS patients and healthy subjects, we could investigate the ability of iNKT cells to produce different cytokines by using intracellular staining and acoustic flow cytometry. Since, in this set of analyses, the age of patients and healthy subjects was matched, we used one group of control donors. The strategy to identify the production of up to four cytokines by different subsets of iNKT cells after 4-h stimulation is shown in Figure 3. Doublets and fluorochrome aggregates were removed by electronic gates, and living T lymphocytes were identified on the basis of the expression of CD3 and negativity for live–dead probe, and then iNKT cells expressing CD4 or CD8 were recognized. In each subset defined by CD4 or CD8 expression, or in double negative (DN) cells, the intracellular presence of TNF-α, IFN-γ, IL-4, or IL-17 was identified.

We could measure both the “total” response, that is, the sum of all cells positive for at least one marker (this provides the overall frequency of responding cells) and the “qualitative” response, which describes the contribution of each functional pattern to the total specific response. Surprisingly, we pointed out that iNKT cells of patients and controls did not display any significant polyfunctionality feature except for the co-production of IFN-γ and TNF-α among CD4−CD8− iNKT cells of RR patients treated with different therapies. Cells were characterized by the predominant expression of Th1 and Th17 cytokines that, however, was not simultaneous to that of other molecules. For this reason, we refer to the total production of each cytokine.

### Among Untreated Patients, SP Patients Displayed Higher Levels of iNKT Cells Able to Produce IL-17

**Figure 4:**
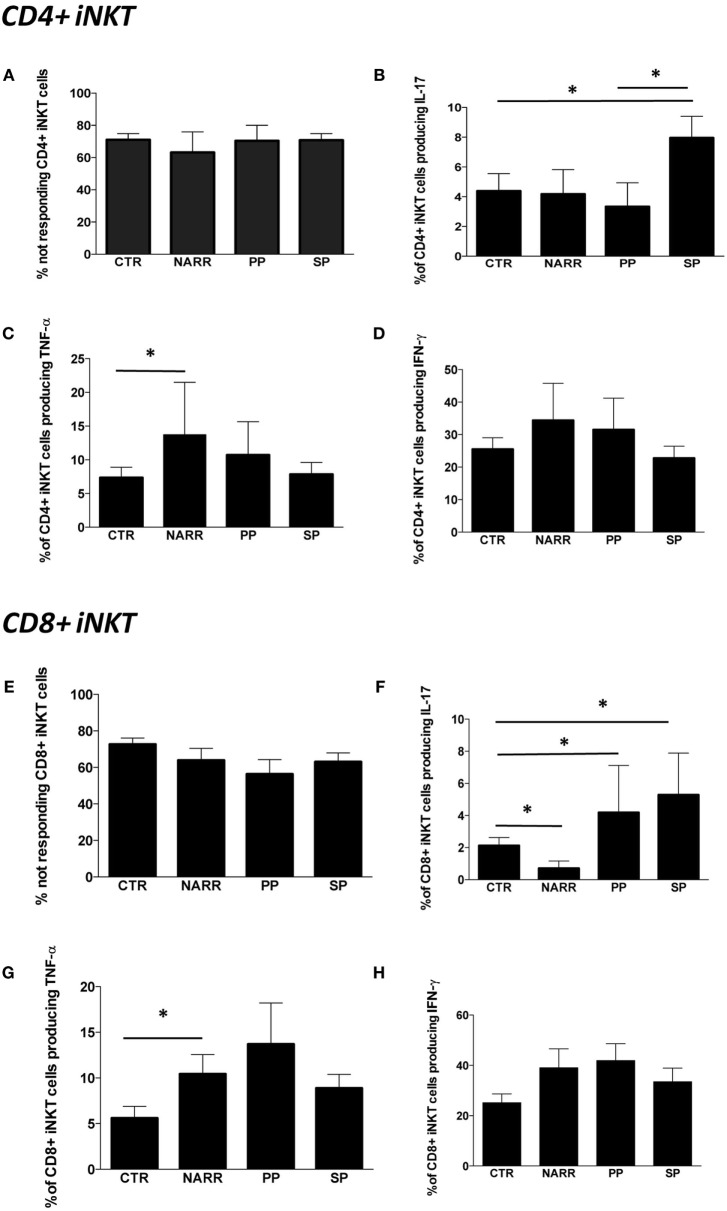
**Cytokine production by different subpopulations of iNKT cells after stimulation**. **(A)** Percentage of CD4+ iNKT cells that did not produce any cytokine after *in vitro* stimulation (not responding); **(B)** total IL-17 production by CD4+ iNKT cells; **(C)** total TNF-α production by CD4+ iNKT cells; **(D)** total IFN-γ production by CD4+ iNKT cells; **(E)** percentage of not responding CD8+ iNKT cells; **(F)** total IL-17 production by CD8+ iNKT cells; **(G)** total TNF-α production by CD8+ iNKT cells; **(H)** total IFN-γ production by CD8+ iNKT cells. The functional analysis was carried out on: 5 patients with not-active RR, 4 with PP, 12 with SP and 26 CTR. Bar graphs indicate mean ± SEM; *p < 0.05.

The percentage of not responding CD4+ iNKT cells after stimulus was similar among different forms of MS patients and controls, and was approximately 70% (Figure 4A). The remaining 30% of iNKT cells expressing CD4 were able to produce Th1 and Th17 cytokines. In particular, SP patients displayed higher levels of CD4+ iNKT cells producing IL-17 if compared to CTR and to PP patients (Figure 4B). NARR patients displayed a higher percentage of CD4+ iNKT cells producing TNF-α if compared to CTR (Figure 4C). No differences were found in the percentage of cells producing IFN-γ (Figure 4D).

The percentage of CD8+ iNKT cells able to produce cytokines after *in vitro* stimulation was similar in all forms of MS (Figure 4E). However, progressive patients (both PP and SP) were characterized by higher levels of CD8+ iNKT cells that produced IL-17 if compared to controls, while NARR patients displayed lower percentages of these cells, even when compared to controls (Figure 4F). Moreover, NARR patients showed high levels of CD8+ iNKT cells, able to produce TNF-α if compared to controls (Figure 4G), while also in the case of CD8+ T cells, no differences among groups were found in the production of IFN-γ (Figure 4H).

### Natalizumab Turns Off Pro-inflammatory Cytokine Production by iNKT Cells

**Figure 5:**
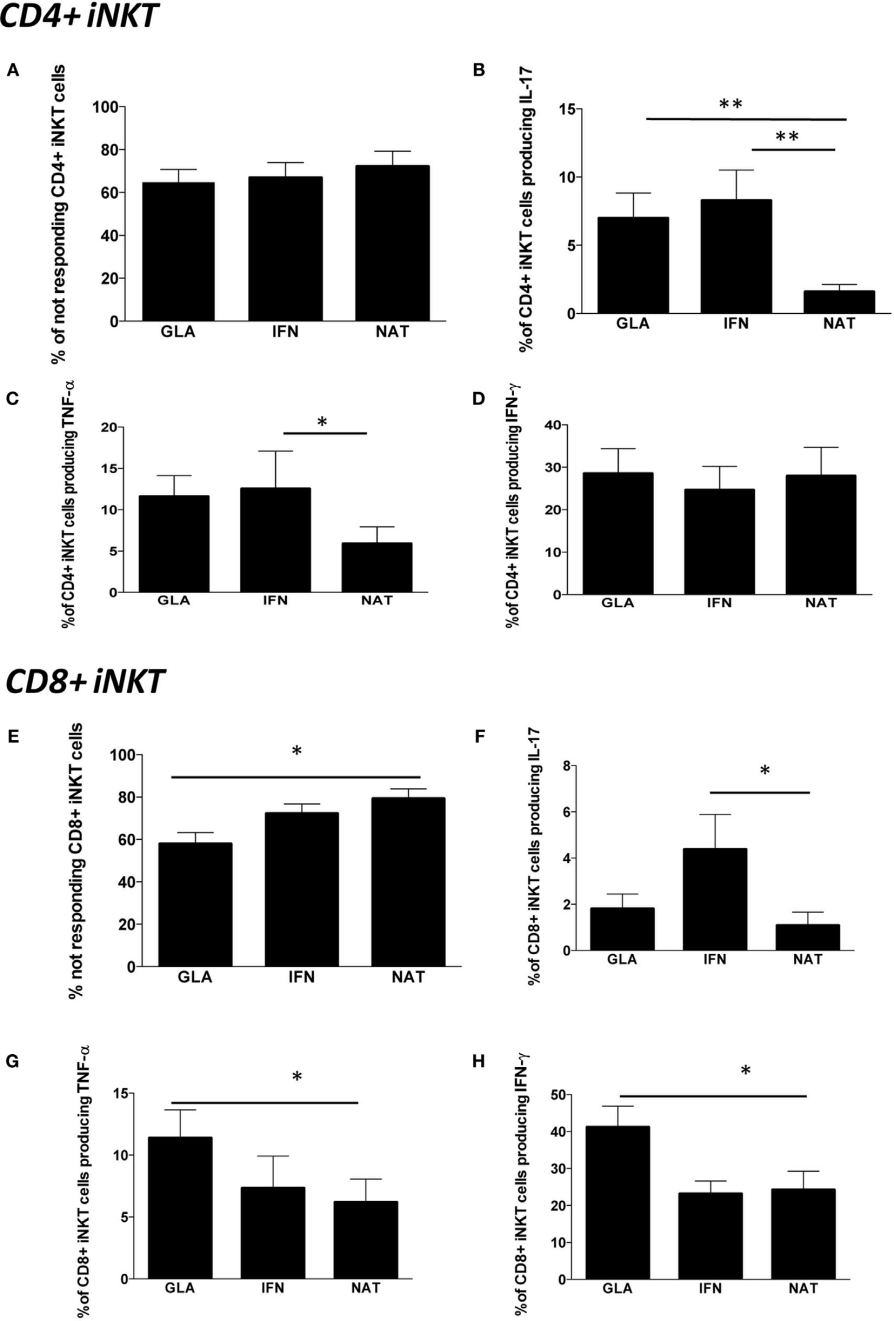

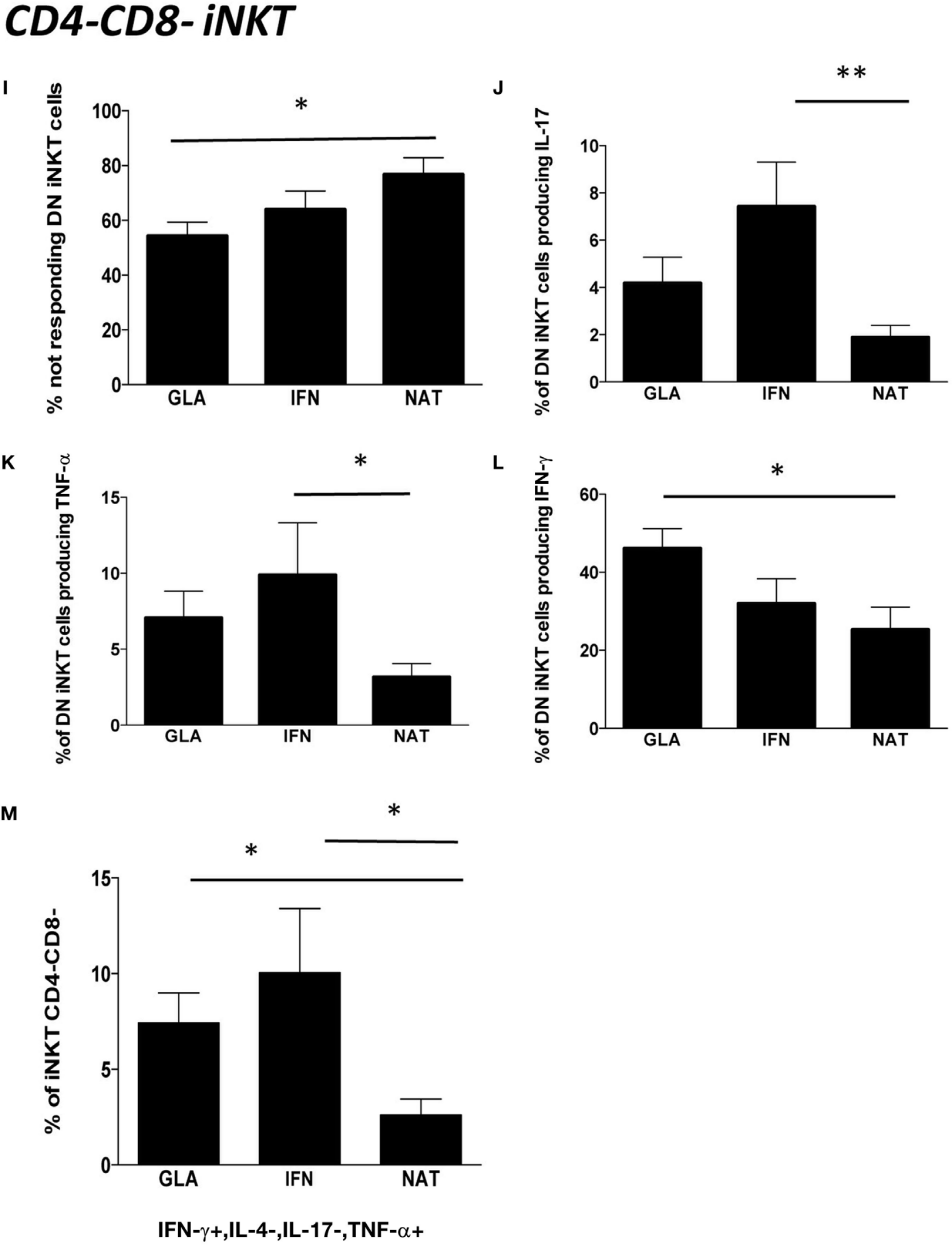
**Cytokine production by different subpopulations of iNKT cells in RR patients treated with different immunomodulatory drugs**. **(A)** Percentage of CD4+ iNKT cells not responding after *in vitro* stimulation; **(B)** total IL-17 production by CD4+ iNKT cells; **(C)** total TNF-α production by CD4+ iNKT cells; **(D)** total IFN-γ production by CD4+ iNKT cells; **(E)** percentage of not responding CD8+ iNKT cells; **(F)** total IL-17 production by CD8+ iNKT cells; **(G)** total TNF-α production by CD8+ iNKT cells; **(H)** total IFN-γ production by CD8+ iNKT cells; **(I)** percentage of not responding CD4−CD8− iNKT cells; **(J)** total IL-17 production by CD4−CD8− iNKT cells; **(K)** total TNF-α production by CD4−CD8− iNKT cells; **(L)** total IFN-γ production by CD4−CD8− iNKT cells; **(M)** polyfunctionality of CD4−CD8− iNKT cells after *in vitro* stimulation. Bar graphs indicate mean ± SEM; *p < 0.05; **p < 0.01. The functional analysis was carried out on: 13 RR patients treated with NAT, 11 with GA, and 12 with IFN.

Last, we investigated the modulatory effects of different immunomodulatory treatments on cytokine production by iNKT cells in patients treated with GA, IFN, or NAT for at least 6 months. The overall percentage of CD4+ iNKT cells responding to a potent stimulation was similar in all the groups (Figure 5A). However, patients treated with NAT displayed lower levels of CD4+ iNKT cells producing IL-17 if compared to all other treatments (Figure 5B), along with lower levels of TNF-α (Figure 5C). No differences were found in the production of IFN-γ (Figure 5D).

Nearly, 80% of CD8+ iNKT of patients treated with NAT did not respond to the *in vitro* stimulation, a percentage that was higher than that found in patients treated with GA or IFN (Figure 5E). Accordingly, patients treated with NAT displayed the lowest percentage of CD8+ iNKT cells able to produce IL-17 (Figure 5F), TNF-α (Figure 5G), and IFN-γ (Figure 5H).

As far as DN CD4−, CD8− iNKT cells are concerned, patients treated with NAT showed the highest percentage of cells unable to produce cytokines (Figure 5I). They also displayed the lowest percentage of cells able to produce IL-17 (Figure 5J), TNF-α (Figure 5K), and IFN-γ (Figure 5L). Considering the polyfunctionality of these cells, we found that DN iNKT cells able to produce IFN-γ and TNF-α, but not IL-4 or IL-17, were decreased in patients treated with NAT (Figure 5M). Using the software SPICE, no significant changes were noted as far as other combinations are concerned (not shown).

## Discussion

### Changes in iNKT Cell Subsets

In the field of immune-mediated diseases, the importance of iNKT cells was first recognized through the observation that mice lacking CD1d or iNKT cells were predisposed to develop several autoimmune diseases (34), but few data are present as far as the role of iNKT cells in the pathogenesis of MS is concerned. Thus, the purpose of the present study was to finely analyze the phenotypic and functional characteristics of peripheral iNKT cells in 165 MS patients compared to 55 healthy subjects.

No significant differences were found in the overall percentage and in the absolute number of total iNKT cells in patients with different forms of MS; nevertheless, significant changes took place among different iNKT cell subpopulations. Moreover, functional differences not only among patients with different MS forms but also among those treated with different disease-modifying drugs (DMDs) have been detected.

Previous studies reported that MS patients showed decreases (20, 35) or increases in the number of iNKT cells (15) compared to controls, while no clear differences between MS patients and controls have been found in the present study. These discrepancies could be due to several reasons, including the different panels of monoclonal antibodies used for the flow cytometric analysis and the use of different strategies for their identification. In fact, our approach was based upon the strategy of detection of rare cells: we analyzed a minimum of ten million events per sample and used fresh blood, against 100,000–200,000 events acquired in previous studies. Moreover, previous studies enrolled small cohorts of patients, while our study not only included a much higher number of patients but patient groups were also more homogeneous, since we carefully considered patients’ disease activity profile, treatment regimen, and age.

We found that, in RR patients, the percentage of iNKT cells was not affected by DMDs. A previous study reported that treatment with IFN significantly increased the percentage of peripheral Vα24+ T cells (36). However, the cohort of IFN-treated and untreated patients was relatively small (eight treated vs. four non-treated patients).

Regarding different iNKT cell subsets, in the present study, ARR patients and PP patients showed a higher percentage of iNKT cells expressing CD4+ compared to controls. SP patients showed the same trend, even though statistical significance was not reached. These findings suggest that the active phase of MS, as well as its progression, seems to be characterized by a sustained presence of CD4+ iNKT cells, which probably contribute to the presence and/or to the persistence of an inflammatory status.

We found that NARR patients displayed a higher percentage of CD4+ iNKT cells expressing CD161 if compared to SP patients. Even though the meaning of CD161 expression by iNKT cells remains to be fully elucidated, it is widely accepted that CD161 is involved in the regulation of NKT cell function and, indeed, triggering of CD161 enhances NKT cell proliferation (37). Furthermore, the expression of CD161 is associated with cells expressing a memory phenotype and has been related to the secretion of IL-17, a potent pro-inflammatory cytokine with a recently reported role in progressive MS patients (38).

### Functional Characteristics of iNKT Cells

We found that iNKT cells from patients with different forms of MS were able to respond to a potent stimulation, and they mainly produced Th1 and Th17 cytokines, such as TNF-α, IFN-γ, and IL-17. Previous studies regarding such cells in RR patients showed that patients with “inactive” MS, compared with those during a clinical relapse, exhibit a prevailing Th2 phenotype, indicating indirectly that this immunosuppressive phenotype could be absent in patients who are in the active phase of the disease (35). Our data on the functionality of these cells, in particular on CD4+ iNKT cells producing IL-17, support this hypothesis, and suggest that this phenotype could be related with inflammation in active and progressive disease.

Interestingly, iNKT cells were not polyfunctional in most cases. SP patients displayed a Th17 pattern of cytokine production and a higher percentage of CD4+ iNKT cells producing IL-17 compared to controls. Furthermore, progressive patients (both PP or SP), who displayed higher levels of CD4+ iNKT cells showed higher amounts of CD4+ iNKT cells able to produce TNF-α.

A close association between inflammation and neurodegeneration has been reported in all disease stages of MS (39, 40), and the extent of axonal injury appears to be related with the degree of inflammation (41) and of disability accumulation. Our results in the progressive forms of MS support the hypothesis of a pathogenetic role of inflammation in the development of disease progression. Many previous studies reported increased activation of peripheral blood immune cells from progressive patients, while pathology studies indicate that in SP and PP the inflammation is abundant, widespread and diffuse in CNS, and correlates with axonal damage and disease progression (42). A crucial role of IL-17 and of dysregulation of the IL-23/IL-17 axis in the blood of SP patients, and a correlation between the levels of these pro-inflammatory factors, the lesion load at MRI and brain atrophy have been recently documented (43, 44). Studies in experimental autoimmune encephalitis animal models showed that myelin-reactive Th17 cells induces ectopic follicles in the CNS (45), and this seems particularly intriguing in light of the discovery of lymphoid follicle-like structures in the meninges and in the large perivascular space of CNS of SP patients’ brain specimens, supporting the hypothesis of a compartmentalization of, and sustained inflammation in the CNS of progressive stages of MS (46, 47). Our data on iNKT cells in SP-MS patients support the hypothesis that Th17 responses play a crucial role in the development of the progressive phases of MS (44, 48, 49) maybe, in part, *via* activation of iNKT cells, and the production of Th1 and Th17 cytokines.

### DMDs Influence iNKT Cells

Regarding RR patients treated with DMDs, those treated with NAT displayed a higher, but not significantly so, percentage of CD4+ iNKT cells expressing CD161, a lower production of IL-17 and TNF-α by both CD4+ and CD8+ iNKT cells, compared to patients treated with IFN.

The immunological effects of NAT, one of the latest and most effective therapies (50), on peripheral blood cells of RR patients are still unclear. NAT acts as an antagonist of the receptor VLA-4 beta-integrin by inhibiting the transmigration of autoreactive B and T lymphocytes into the CNS (51). Likely, this is not the only biological action of the drug. According to the AFFIRM trial, in RR patients, treatment with NAT reduced the annualized relapse rate by 68% compared to placebo, caused a 42% reduction of sustained disability progression over 2 years, and had a strong therapeutic effect on MRI disease activity (83% of reduction of new T2 lesions and contrast-enhancing lesions) (52).

Recent studies suggest that NAT induces changes in the phenotype of lymphocytes, which may be caused by direct induction of intracellular signaling events and seem to induce a mild upregulation of pro-inflammatory phenotypes in some patients, without modifying regulatory T cell function (53–55). The changes in the Th1/Th2 paradigm cannot, however, explain the beneficial effect of NAT, and it was suggested that it might be linked to the blockade of migration in the CNS and to the subsequent accumulation of activated lymphocytes in the periphery during exposure to the drug.

All these studies have, however, several limitations, including very small patient numbers and different timing of cytokines measurements. To date, to our knowledge, no other studies have focused on peripheral iNKT cells of MS patients treated with NAT. The possible role of cytokine profile changes induced by NAT treatment remains largely controversial and requires further investigation. In any case, our results on the polyfunctionality of iNKT cells indicate that NAT seems to inhibit their pro-inflammatory action.

## Conclusion

This study has characterized iNKT phenotype and functional activities in different forms of MS and during treatment with different DMDs. Our results indicate that the SP form is characterized by activation of iNKT cells in the peripheral blood, which are skewed toward the production of Th1 and Th17 cytokines, supporting an important role of inflammation also in the progressive phases of the disease. Moreover, we suggest that DMDs seem to modulate iNKT cell function.

No gross differences in iNKT percentages or in their number have been found in the different forms of disease or in response to different therapies. The more important findings were related to the function of these cells and to their ability of modulating and impacting cytokine homeostasis. Recent data indicate that iNKT cells differentiating out of the intestine do not produce IFN-γ (56), and that, in general, the production of cytokines by iNKT cells is governed by their localization (57). Moreover, cytokine and cell profiles in the periphery may differ, and be even opposite, to those in the CNS. To date, there is no data on the behavior of such cells in the context of the CNS. Further studies are, thus, needed to examine whether the iNKT-cell pro-inflammatory profile is present in different tissues and organs of MS patients, and whether these cells may exert a harmful effect.

## Author Contributions

SDB performed experiments and participated in the design of the study; EB performed experiments; AS, DF, FV, and PS performed the patients’ selection; MN, LG, MP, and CG performed statistical analysis; PS and AC designed the study and wrote the manuscript. All the authors read and approved the final manuscript.

## Conflict of Interest Statement

The authors declare that the research was conducted in the absence of any commercial or financial relationships that could be construed as a potential conflict of interest.
